# Silencing ARAF Suppresses the Malignant Phenotypes of Gallbladder Cancer Cells

**DOI:** 10.1155/2020/3235786

**Published:** 2020-08-18

**Authors:** Weiguo Lin, Chenhao Tong, Weiguang Zhang, Wenda Cen, Yali Wang, Jiandong Li, Zhiyang Zhu, Jianhua Yu, Baochun Lu

**Affiliations:** ^1^Department of Hepatobiliary Surgery, Shaoxing People's Hospital (Shaoxing Hospital, Zhejiang University School of Medicine), Shaoxing, China; ^2^Department of Urinary Surgery, Ruian People's Hospital, Wenzhou, China; ^3^Department of Molecular Medicine and Clinical Laboratory, Shaoxing Second Hospital, Shaoxing, China; ^4^Shaoxing University School of Medicine, Shaoxing, China

## Abstract

ARAF is a member of the RAF kinase family that is necessary for mitogen-activated protein kinase (MAPK) activation in various malignancies, including lung, colorectal, pancreatic, and breast cancers. As the most common biliary tract tumor, gallbladder cancer (GBC) seriously harms human health while the function of ARAF in GBC remains elusive. Here, we found that ARAF expression was upregulated in gallbladder cancer tissues. *In vitro*, ARAF silencing mediated by RNA interference effectively inhibited cell proliferation, colony formation, migration, and invasion of GBC cells. Moreover, knocking down ARAF suppressed tumor growth *in vivo*. Our results indicated that ARAF functions as an oncogene in GBC and, thus, could be a potential therapeutic target for GBC.

## 1. Introduction

Gallbladder cancer (GBC) is an aggressive malignancy of the biliary tract that originates from the gallbladder and cystic duct mucosal epithelia [1]. As the most common biliary tract cancer, GBC accounts for 80%-95% of all biliary malignancies and has a dismal prognosis [2, 3]. The recent optimization of medical auxiliary examinations and the widespread application of laparoscopic cholecystectomy have significantly increased the detection rate of gallbladder cancer; however, its prognosis has not improved because of late-stage diagnoses, high recurrence rates, and metastatic features [4]. Although surgical resection remains the most effective treatment for GBC, most patients are diagnosed with advanced-stage disease, meaning they are not candidates for surgery [5, 6]. What is worse, GBC has extremely poor sensitivity to radiotherapy and chemotherapy. Therefore, clearing the underlying molecular mechanisms of GBC tumorigenesis and metastasis will provide a theoretical basis for improving its diagnosis and treatment.

Located on human chromosome band Xp11.3, ARAF belongs to the serine/threonine protein kinase gene family [7]. Similar to other RAF family members, ARAF transduces mitogen-activated protein kinase (MAPK) signaling from RAS to MEK and ERK, thus promoting cell proliferation, differentiation, migration, and survival [8, 9]. The RAS-RAF-MEK-ERK cascade is considered to be a therapeutic target in various cancers [10, 11].

Early studies on the RAF family focused on B-Raf and C-Raf kinases, resulting in little understanding of the biological function of ARAF. Recent studies focused on the role of ARAF in tumor progression have made significant impact on the field. Early cancer sequencing studies identified high-copy number gains as well as oncogenic driver mutations in ARAF in lung cancer patients [12]. In 2014, a study demonstrated that ARAF was required for MAPK activation in a variety of cancer types (e.g., colorectal, pancreatic, and breast cancers) and further verified that ARAF enhanced the migration and invasive ability of these tumor cells [13]. Other studies reported that ARAF mutations could drive lung cancer and that the RAF-targeted kinase inhibitor sorafenib improved the prognosis of advanced lung cancer patients, thus providing a new opportunity for lung cancer treatment [14]. These findings suggested that ARAF could be a therapeutic target in numerous cancers. However, the functional role of ARAF in GBC has not been verified.

Here, we explored the functional roles of ARAF in relation to GBC tumorigenesis and progression. As shown in our results, both ARAF mRNA and its encoding protein were overexpressed in GBC compared with nontumoral tissues. After the expression level of ARAF gene was downregulated by RNA interference technology, the tumor phenotype of gallbladder cancer cells was considerably affected both *in vivo* and *in vitro*, which showed that the cell proliferation, metastasis, and other abilities were weakened. Therefore, we believe that ARAF promotes the development of GBC and regulates its growth and metastasis.

## 2. Materials and Methods

### 2.1. Clinical Tissue Samples

GBC and normal gallbladder tissues were obtained at Shaoxing People's Hospital. All patients signed informed consent documents before inclusion in the study. Informed consent document and tissue acquisition protocol were approved by the Ethics Committee of Shaoxing People's Hospital (Shaoxing, China). Cancer tissues were collected from GBC patients, while nontumoral tissues were harvested from patients with gallbladder polyps. Fresh tissues were stored in liquid nitrogen prior to RNA and protein extraction.

### 2.2. Cell Culture

The GBC cell line GBC-SD was purchased from the Chinese Academy of Sciences Shanghai Branch Cell Bank (Shanghai, China), and the SGC-996 cell line was obtained from Dr. Ying-Bin Liu's lab at Xin Hua Hospital Affiliated to Shanghai Jiao Tong University School of Medicine, China. Both cell lines were cultured in RPMI-1640 medium (cat. no. GNM-31800-S; USEN Biological Technology Co., Ltd., Shanghai, China) with 10% fetal bovine serum (FBS; cat. no. 16140071; Gibco; Thermo Fisher Scientific, Inc., Waltham, MA, USA), 100 IU/ml penicillin, and 100 *μ*g/ml streptomycin in a 37°C incubator with 5% CO_2_.

### 2.3. siRNA Transfection

To downregulate ARAF expression in GBC cell lines, ARAF-targeting siRNA (5′-GGGATGGCATGAGTGTCTA-3′) was purchased from RiboBio (Guangzhou, China). Control siRNA was also obtained from RiboBio, and the control sequence was not public. Before transfection, cells were seeded into dishes at 50%–60% confluence. Transfection was performed using Lipofectamine 2000 (Invitrogen, Carlsbad, CA, USA) according to the manufacturer's protocol.

### 2.4. RNA Extraction and RT-qPCR

TRIzol (Invitrogen) was used to isolate total RNA from tissues or cells. Then, RNA was reverse transcribed into cDNA using the PrimeScript Reagent Kit (Takara, Shiga, Japan) according to the manufacturer's instruction. RT-qPCR was performed using the TB Green Kit (Takara) on an ABI 7500 Real-time PCR system (Applied Biosystems, Foster City, CA, USA). GAPDH was used as an endogenous control. Relative ARAF mRNA level was determined by the 2^-*ΔΔ*Ct^ method [15]. The primer sequences are listed as follows: *ARAF* forward 5′-CCCACATTCCAAGTCACCAGCA-3′ and reverse 5′-CCTCCCAGTAATAGCCTGAGTC-3′ and GAPDH forward 5′-GTCTCCTCTGACTTCAACAGCG-3′ and reverse 5′-ACCACCCTGTTGCTGTAGCCAA-3′.

### 2.5. Western Blot Analysis

Western blotting was used to detect ARAF protein levels in tissues and cell lines. RIPA lysis buffer containing 1% phenylmethylsulfonyl fluoride (Beyotime Institute of Biotechnology, Nantong, China) was used to extract the total protein from tissues and cells. The protein concentration was determined using the BSA method (Beyotime Institute of Biotechnology). Briefly, 30 *μ*g protein was loaded onto 10% SDS–PAGE gels, electrophoresed, and transferred onto to polyvinylidene fluoride membranes, which were blocked by 5% skim milk powder in TBST, incubated with primary antibodies overnight, and then with horseradish peroxidase-conjugated secondary antibodies (cat. nos. A0208 and A0216; Beyotime Institute of Biotechnology) at 1 : 10000 dilution for 2 h at room temperature. Primary antibodies against ARAF (dilution 1 : 1000; cat. no. 4432) and phospho-p44/42 MAPK (Erk1/2, Thr202/Tyr204, dilution 1 : 1000; cat. no. 4370) were purchased from Cell Signaling Technology (Danvers, MA, USA). Primary antibodies against PCNA (dilution 1 : 1000; cat. no. 60097-1-Ig), cyclin D1 (dilution 1 : 1000; cat. no. 26939-1-AP), and *β*-actin (dilution 1 : 1000; cat. no. 20536-1-AP) were purchased from Proteintech (Rosemont, IL, USA). *β*-Actin was used as the endogenous control. Immunoreactive bands were visualized using a chemiluminescence solution (Beyotime Institute of Biotechnology).

### 2.6. Cell Proliferation Assays

Both GBC cell lines were transfected with ARAF siRNA or siRNA control and, 6 h after transfection, were seeded into 96-well plates at 2000 cells per well. Every 24 h, cell growth was evaluated using Cell Counting Kit-8 (Beyotime Institute of Biotechnology). According to the manufacturer's protocol, 10 *μ*l of CCK-8 reagent was added to each well and incubated for 2 h. Then, cell viability was measured with an enzyme-labeling instrument (BioTek, Winooski, VT, USA) at 450 nm.

### 2.7. Colony Formation Assays

After transfection, 200 cells were seeded into 35 mm dishes and then cultured for 2 weeks. After fixation with 4% paraformaldehyde and staining with 0.1% crystal violet solution, colonies of >50 cells were counted.

### 2.8. Wound Healing Assays

Cells were seeded and grown to confluence on 35 mm cell dishes. Six hours posttransfection, a 10 *μ*l pipette tip was used to scratch the confluent monolayers. Cells were then cultured in serum-free medium (inhibiting cell proliferation), and after 48 h, images of the wounds were captured at 100x magnification. Wound healing was quantified as the average linear speed of the wound edges. The scratch area was calculated by ImageJ software, and the cell mobility was calculated by the following formula: the cell mobility = (*T*_0_ − *T*_48_)/*T*_0_ × 100%.

### 2.9. Transwell Assays

Briefly, 2 × 10^4^ transfected cells per 100 *μ*l in serum-free medium were added to the upper chamber of the insert (Corning Inc., Corning, NY, USA), while the lower chamber was filled with 0.5 ml of medium containing 20% FBS. Chambers were incubated for 24 h, and then, invasive cells were fixed with 4% paraformaldehyde and stained with 0.1% crystal violet. Four fields of view were taken under a 100x optical microscope, and the number of cells entering the lower chamber in each field was calculated and averaged.

### 2.10. Xenograft Formation Assays

All procedures were approved by the Ethics Committee of Shaoxing People's Hospital and conformed to the Care and Use of Laboratory Animals guide published by the US National Institutes of Health (NIH Publication No. 85-23, revised 1996). Six-week-old athymic nude mice were supplied by Shanghai SLAC Laboratory Animal Co., Ltd. (Shanghai, China). The mice had ad libitum access to food and water and were maintained at 20°C, with 50% humidity under 12 : 12-h light-dark cycles. Then, 2 × 10^6^ cells were suspended in 0.2 ml PBS and subcutaneously injected into the back of nude mice. siRNA was injected intratumorally in a volume of 100 *μ*l once every 3 days. At regular intervals, tumor sizes were measured, and tumor volumes were calculated according to the following formula: volume = 1/2 × length × width^2^ [16]. The mice with GBC-SD cells or SGC-996 cells were sacrificed via cervical dislocation under isoflurane anesthesia after 42 days or 24 days, respectively. Finally, all tumor specimens were collected and weighed.

### 2.11. Statistical Analysis

All experiments were repeated at least three times, and data are presented as the means ± SD. Student's *t*-test was used to determine statistical significance between two groups. One-way ANOVA followed by the Tukey–Kramer adjustment was used to examine differences among multiple groups. All statistical analyses were conducted using SPSS v21.0 (IBM, Armonk, NY, USA), and *P* < 0.05 was considered statistically significant.

## 3. Results

### 3.1. ARAF Expression Is Upregulated in GBC Tissues

**Figure 1:**
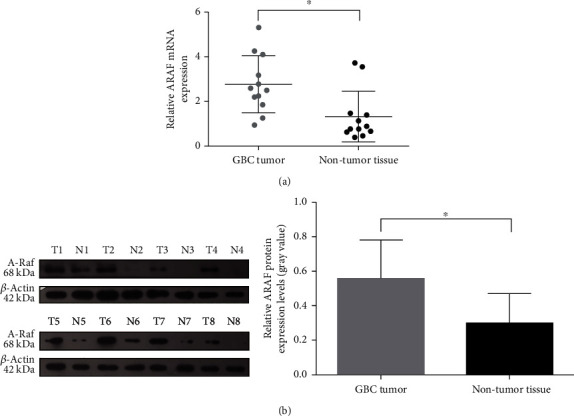
ARAF expression is upregulated in GBC tissues. (a) Detection of the mRNA levels of ARAF in GBC tissue samples. (b) Detection of the protein levels of ARAF in GBC tissue samples. Left panel: representative Western blot results were shown. Right panel: summary of the results. ∗*P* < 0.05.

To compare ARAF mRNA expression between GBC and nontumoral samples, RT-qPCR was performed, and the average ARAF mRNA expression of 12 nontumoral tissues was defined as the baseline expression of normal tissues. As shown in Figure 1(a), ARAF mRNA expression was significantly higher in GBC tissues than that in nontumoral tissues according to the results of RT-qPCR (Figure 1(a)). Additionally, ARAF protein was also significantly increased in GBC (Figure 1(b)).

### 3.2. Silencing ARAF Inhibits GBC Cell Proliferation

**Figure 2:**
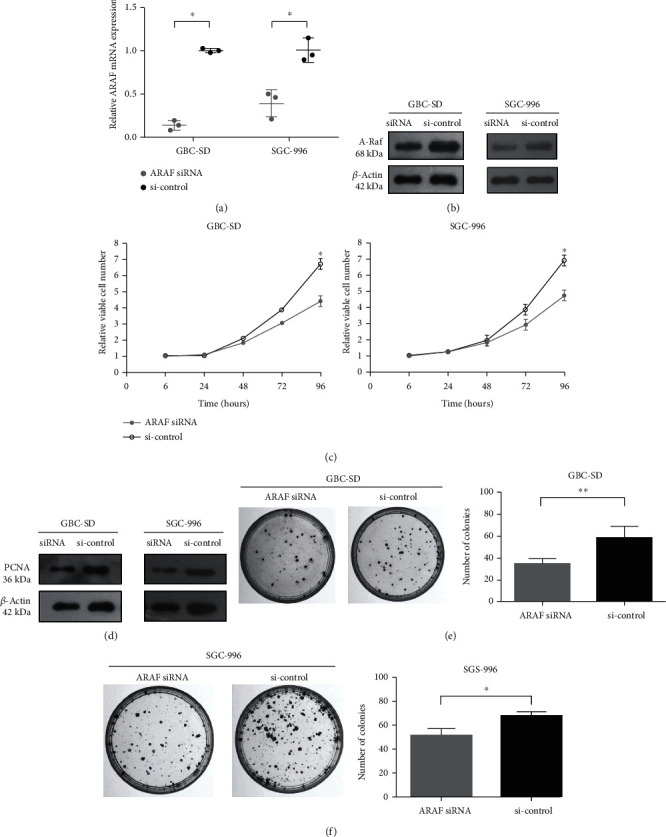
Downregulated expression of ARAF inhibits GBC cell proliferation. (a, b) RT-qPCR and western blot analysis were performed to confirm the effect of ARAF silencing. (c) Cell proliferation assays of ARAF silencing GBC cells. (d) Detection of the protein levels of PCNA in ARAF siRNA and siRNA control groups. (e, f) Knockdown of ARAF inhibited colony formation of both GBC cell lines. Colonies were counted only if a single clone contained more than 50 cells. ^∗∗^*P* < 0.01.

ARAF knockdown was employed to study its function in GBC cells. We examined ARAF expression in GBC-SD and SGC-996 cells transfected with ARAF siRNA or siRNA control. After siRNA transfection, ARAF mRNA and protein levels were both significantly lower than controls (Figures 2(a) and 2(b)). It was demonstrated that the ARAF siRNA successfully silenced endogenous ARAF in GBC cells. Inhibiting the rapid growing of cancer cells is an important way to treat cancers. As shown in Figure 2(c), the CCK-8 assays showed that ARAF siRNA inhibited the proliferation of different GBC cell lines, including GBC-SD cells and SGC-996 cells. Interestingly, PCNA, a reliable indicator of cell proliferation, was also higher in the control siRNA group compared with ARAF knockdown (Figure 2(d)). Furthermore, colony formation was significantly reduced in the ARAF siRNA group, compared with controls (Figures 2(e) and 2(f)).

### 3.3. Silencing ARAF Inhibits the Migration and Invasion of GBC Cells

**Figure 3:**
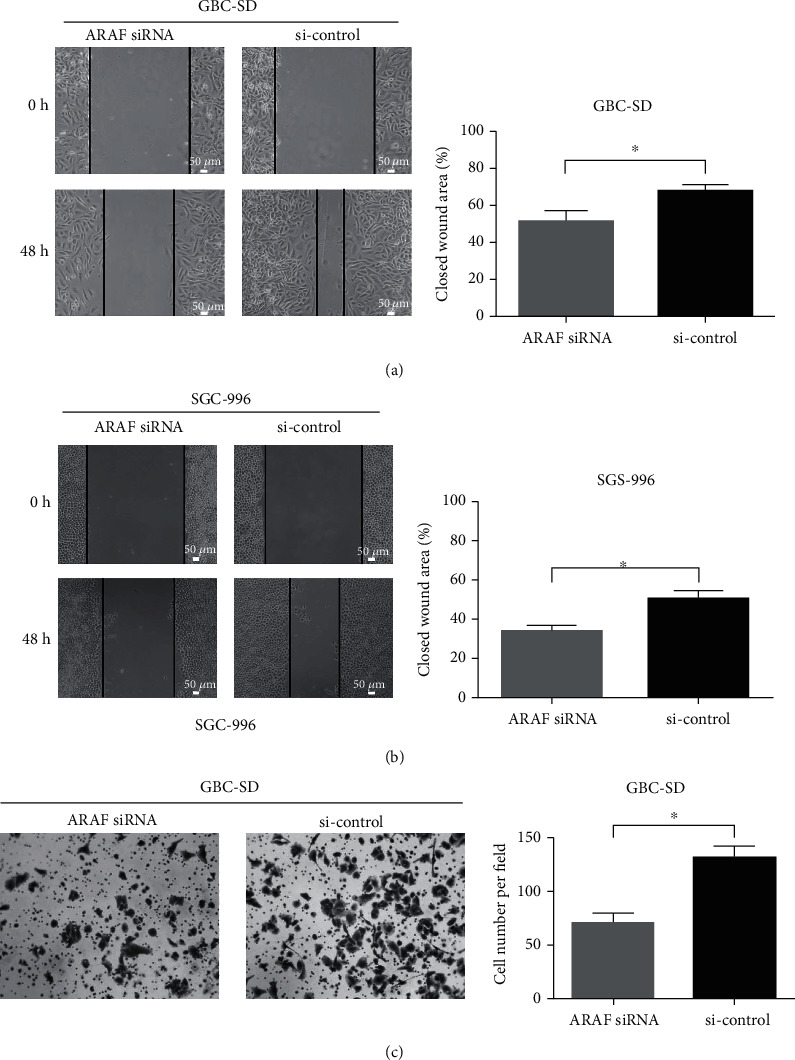
ARAF promotes migration and invasion of GBC cells. (a, b) Wound healing assays were utilized to identify the role of ARAF in migration. (c) Results of transwell assays of GBC-SD cells transfected with ARAF siRNA or siRNA control. ^∗^*P* < 0.05.

Metastasis is the most discouraging phenomenon in cancers and is also an important factor which leads the patients with the late stage of GBC lose the operation opportunity. The role of ARAF on the migration and invasion of GBC cells was further explored by wound healing and transwell assays. As shown in Figures 3(a) and 3(b), ARAF knockdown significantly attenuated cell migration compared with controls. Because we found that the invasiveness of SGC-996 cells is too poor to use for transwell assays, GBC-SD cells were employed to investigate the role of ARAF on cell invasion. We found that the invasion of GBC-SD cells was remarkably suppressed after ARAF was knockdown (Figure 3(c)). These results indicate that silencing ARAF inhibits the migration and invasion of GBC cells.

### 3.4. Silencing ARAF Suppresses Xenograft Tumor Growth In Vivo

**Figure 4:**
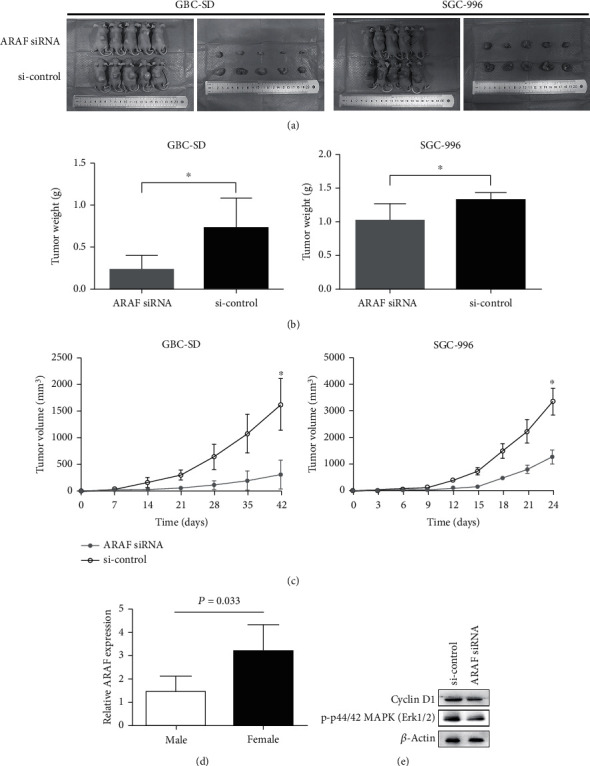
Silencing ARAF suppresses xenograft tumor growth *in vivo.* (a) The photograph of nude mice and xenograft tumors. (b, c) The weight and the growth curve of xenograft tumors were measured. (d) Different expression level of ARAF between male and female GBC patients. (e) Phosphorylation of Erk and cyclin D1 decreased when ARAF was knocked down in GBC cells. ^∗^*P* < 0.05, compared with the si-control group.

Nude mouse xenograft formation assays were performed to investigate the biological significance of silencing ARAF in GBC by subcutaneously injecting GBC-SD and SGC-996 cells. Both the tumor volume and weight of nude mice in the ARAF silencing group were significantly reduced, compared with the control group (Figures 4(a)–4(c)). It indicates that silencing ARAF also effectively suppresses tumor growth *in vivo*, consistent with the results *in vitro*.

## 4. Discussion

GBC is the seventh most common tumor worldwide and has a terrible prognosis [17]. The main reasons for its poor prognosis are late diagnosis, early metastasis, and limited therapeutic options, which make it urgently necessary to uncover the molecular mechanism of GBC.

Among patients with gallbladder cancer, it is clear that the proportion of women is significantly higher than that of men. Given the gender differences in gallbladder cancer prevalence, we tried to find out protooncogenes on the X chromosome or antioncogenes on the Y chromosome at the beginning of our study. After a literature search and preliminary experiments, the ARAF gene, located on the X chromosome, got our attention. Interestingly, the expression of ARAF was significantly higher in female patients, compared with that in male patients (Figure 4(d)). Given the potential association between the aberrant ARAF expression and the sexual dimorphism of GBC, we thought ARAF may be an oncogene which is more worth to be studied.

As a new star of the family of Raf kinases, ARAF plays an important role in the regulation of many cellular functions, including differentiation, cell proliferation, and transformation [18]. In the mouse experiment with gene knockout of ARAF, mouse embryonic fibroblasts delayed entering the S phase of cell cycle, indicating that ARAF maintained the progress of cell cycle [19]. ARAF also has been shown to play an important role in the proliferation of vascular smooth muscle while inhibiting the activity of Raf kinase could be used as a treatment for vascular hyperplastic diseases [20]. Many kinases which have regulating function during the process of embryonic developing always are potential protooncogenes, including ARAF.

Previous investigation of the protooncogene ARAF demonstrated that ARAF played an obligatory role in promoting MAPK activity as a kinase [13]. MAPK signaling, represented by the phosphorylation of Erk, plays a key role during the cell proliferation, migration, and invasion in various cancers. To clear whether silencing ARAF suppresses the malignant phenotypes of GBC cells through regulating MAPK signaling, phosphorylation of Erk was examined in our study. We found that the phosphorylation of Erk was significantly inhibited in GBC cells when ARAF was downregulated (Figure 4(e)). Our results showed that the silencing of ARAF could produce an inhibitory effect on GBC cell proliferation and colony formation. Coincidentally, a previous study on murine embryonic stem cells also revealed that ARAF is required for Erk activation and involved in the growth and colony formation [21]. More importantly, silencing ARAF limited the growth of xenograft tumors in nude mice. Cyclin D1 is induced by Raf/MAPK/ERK cascade and plays a key role during proliferation in various cancers [22, 23]. Just like the fact that the phosphorylation of Erk was inhibited, the same decreasing trend of cyclin D1 expression was observed after ARAF was knocked down (Figure 4(e)). Our results also demonstrated that the silencing ARAF impaired the migration and invasion of GBC cells. Interestingly, a previous work about trophoblasts reported that ARAF-mediated activation of the integrin/Erk signaling pathway promotes trophoblast migration and invasion [24]. Taken together, ARAF silencing suppresses the malignant phenotypes of gallbladder cancer cells, and the mechanism may be associated with regulating Erk/cyclin D1 axis.

In conclusion, our results demonstrate that ARAF expression is highly expressed in human gallbladder cancer, and ARAF silencing has an inhibitory effect on various phenotypes of GBC. Based on these findings, ARAF should be regarded as oncogene in GBC progression. Targeting ARAF therefore represents a potential therapeutic target for GBC.

## Data Availability

Data are available on request.
